# DEPRESSIVE AND EATING DISORDERS IN PATIENTS POST-BARIATRIC SURGERY WITH WEIGHT REGAIN: A DESCRIPTIVE OBSERVATIONAL STUDY

**DOI:** 10.1590/0102-672020230002e1725

**Published:** 2023-03-20

**Authors:** Thiago de Almeida Furtado, Marcelo Gomes Girundi, Cláudio de Oliveira Chiari Campolina, Sofia Cunha Mafra, Alice Marina Osório de Oliveira, Maria Luiza Patrão Dias dos Santos, Sarah Ferreira Lopes, Mariana Alvarenga Freire

**Affiliations:** 1 Nexa Clinics, Bariatric Surgery – Belo Horizonte (MG), Brazil; 2 Interdisciplinary Institute of Health and Obesity, Bariatric Surgery – Belo Horizonte (MG), Brazil; 3 Faculdade Ciências Médicas de Minas Gerais – Belo Horizonte (MG), Brazil; 4 Hospital Universitário Ciências Médicas de Minas Gerais – Belo Horizonte (MG), Brazil.

**Keywords:** Bariatric Surgery, Depressive Disorder, Binge-Eating Disorder, Mood Disorders, Weight Gain, Cirurgia Bariátrica, Transtorno Depressivo, Transtorno da Compulsão Alimentar, Transtornos do Humor, Aumento de Peso

## Abstract

**BACKGROUND:**

Although bariatric surgery is today’s gold standard treatment for obesity, weight regain affects the success rate of the procedure. Recent studies have identified psychiatric and neurological factors as possible causes.

**AIMS:**

The aim of this study was to evaluate the influence of psychiatric diseases on the outcome and long-term success of bariatric surgeries and find a weight regain threshold that has an acceptable sensibility to mental health-related issues to be used in research and clinical studies.

**METHODS:**

This is a observational study of bariatric patients submitted to Roux-en-Y bypass or sleeve gastrectomy, with a postoperative time of 2–10 years to access weight regain, depression, and binge-eating disorder.

**RESULTS:**

Of 217 patients studied, 163 were women and 54 were men, with an average postoperative time of 5.2±2.6 years. Weight regain was experienced in 35% of the patients, binge-eating disorder in 24.9%, and depression in 24%. The greater weight before surgery, body mass index (BMI), percentage increase to maximum weight loss, and time postoperatively all have a significant positive correlation with weight regain (p=0.045, p=0.026, p<0.001, and p<0.001, respectively). A significant association between binge-eating disorder, depression, and anxiety with weight regain (p=0.004, p=0.008, and p=0.001, respectively) was found.

**CONCLUSIONS:**

The significant weight regain rates with significant impact on psychiatric disorders highlight the need for continuous postoperative monitoring focused on the psychiatric aspects of obesity to aid surgeries’ long-term success.

## RESUMO

**INTRODUÇÃO:**

Embora a cirurgia bariátrica atualmente é considerada o tratamento padrão ouro para a obesidade, o reganho de peso afeta a taxa de sucesso do procedimento. Estudos recentes identificaram fatores psiquiátricos e neurológicos como possíveis causas.

**OBJETIVOS:**

Avaliar a influência de transtornos psiquiátricos no resultado a longo prazo das cirurgias bariátricas; testar a sensibilidade e correlação das fórmulas de reganho de peso e de seus respectivos pontos de corte para questões relacionadas à saúde mental.

**MÉTODOS:**

Estudo observacional de pacientes pós bariátricos submetidos à *by-pass* em Y de Roux ou gastrectomia vertical com pós-operatório de 2 a10 anos avaliados quanto a reganho de peso, depressão e transtorno da compulsão alimentar.

**RESULTADOS:**

Foram avaliados 217 pacientes, 163 mulheres e 54 homens com pós-operatório de 5,2±2,6 anos. O reganho de peso foi registrado em 35% dos pacientes, o transtorno da compulsão alimentar (TCA) foi encontrado em 24,9% e depressão em 24%. O ganho de peso pré-operatório, o índice de massa corporea (IMC), o aumento percentual para perda máxima de peso e tempo de pós-operatório, apresentaram correlação positiva significativa com o reganho de peso (p=0,045), (p=0,026), (<0,001), (<0,001). Foi encontrada associação significativa entre TCA, depressão e ansiedade com reganho de peso (p=0,004), (p=0,008) e (p=0,001).

**CONCLUSÕES:**

As taxas significativas de reganho de peso associado ao impacto dos transtornos psiquiátricos reforçam a necessidade de acompanhamento pós-operatório contínuo focado nos aspectos psiquiátricos da obesidade, para sucesso do tratamento cirúrgico em longo prazo.

## INTRODUCTION

According to the World Health Organization (WHO), obesity is the most serious health problem in the world, with almost 1.9 billion patients suffering from it. It is a chronic disease with a multifactorial cause: genetic, environmental, socioeconomic, endocrine, metabolic, and psychiatric. Bariatric surgery has been a revolution in the management of obesity since its establishment. The procedure leads to substantial improvements in comorbidities in up to 80% of patients and has been quickly accepted as a useful tool for weight loss, with almost 580,000 procedures performed worldwide every year^1^. Previous studies have shown a higher prevalence of binge-eating disorders (BEDs), depression, and other psychiatric illnesses in obese patients when compared to the overall population^28^.

Despite being considered the gold standard in care, suboptimal rates of weight loss and subsequent recurrence are observed in up to 30% of patients^26^. This proves that the method does not cover all aspects of the disease’s pathogenesis, which explains the long-term limitations of the procedure^26^. Moreover, its worst long-term outcomes, mainly in psychiatric patients, may suggest that surgery alone may not be sufficient for this group of patients.

Additionally, the correlation between weight regain and mood disorders has not yet been described in a clear and consistent way to support such changes in the way surgeons approach obese psychiatric patients or to request the need for more rigorous guidelines^10^. Limitations in the current literature are mainly the lack of standardized guidelines for assessing weight regain, a subjective and not yet standardized psychiatric evaluation for this specific population and length time bias^18^.

Although the preoperative psychiatric evaluation is recommended by existing guidelines, its low rigor and specificity, as well as the lack of perspective by the medical community on the impact of their use on surgical results, lead to a weak and inaccurate adherence to them, especially regarding the psychiatric prerequisites^3^.

The aim of the present study was to investigate the influence of psychiatric diseases on the outcome and long-term success of bariatric surgeries, to determine a weight regain (WR) threshold that has an acceptable sensibility to mental health-related issues to be used in research and clinically, and finally, to suggest the need for bariatrics’ protocols revision so as to include a more rigorous psychiatric evaluation and treatment with comparable importance on the procedure’s outcome to the surgical part itself.

## METHODS

This observational cross-sectional study was approved by the Ethics and Research Committee of the Faculty of Medical Sciences of Minas Gerais (nº 27053519.8.0000.513). Data were collected from medical records and online questionnaires of 3136 patients, submitted to bariatric surgery at two private obesity clinics in Belo Horizonte (MG), after having met the following inclusion criteria: age 18–65 years, submitted to a primary bariatric surgery with either Roux-en-Y gastric bypass (RYGB) or sleeve gastrectomy (SG), from 2010 to 2018.

A pilot study was carried out with 30 patients of the target population of 3136 patients from both bariatric centers to estimate the percentage of WR in the population. With significance of p=0.05, 95% confidence interval and 20% recurrence ratio determined a sample of 229 patients needed for the study.

Out of 3136 patients, 1174 answered the phone and were invited to participate, and 455 agreed to receive the survey via email and authorized the consent form to have their medical records accessed. From these, only 235 answered the questions.

Data searched in the medical records were preoperative weight and body mass index (BMI), psychiatric medications used before surgery, depression, binge eating, and other comorbidities reported by the patient before surgery, and the surgical technique used.

### Instruments

The survey contained the consent form, identification data, questions regarding weight data, the binge-eating disorder scale (BES), and the Hospital Depression Scale (HADS) with questions only from the depression subscale (HADS-D), both diagnoses according to the Diagnostic and Statistical Manual of Mental Disorders (DSM-V)^14,20,26,28^.

The weight data accessed were presurgical weight, nadir weight, time to nadir weight, and current weight. The HADS-D is a self-administration screening scale. The severity of depression is classified as normal (0–7), mild (8–10), moderate (11–15), and severe (16–21), with Cronbach’s internal consistency alpha coefficient of 0.90.

The BES is a self-administered screening tool questionnaire that uses a Likert scale, and the individual can be classified as the studies of community-acquired pneumonia (CAP), employing without CAP (0–17), moderate CAP (18–26), and severe CAP (>27)^14,20,27,28^. Both questionnaires were chosen for their international usage that allows the comparison of the results with those from other countries, as well as for the possibility of classifying the magnitude of the disease in each patient.

### Statistical Analysis

For statistical analysis, the R software version 4.0.3 and a significance level of 5% were considered. Initially, the adherence of data to the normal parameters was accessed. Numerical variables were assessed by the chi-square and Fisher’s exact tests, and the comparison between groups was assessed by the Mann-Whitney U test.

The categorical variables were presented as absolute and relative frequencies and the numerical variables as mean ± standard deviation and median (1st quartile-3rd quartile). From 235 patients’ samples, 2 were excluded for answering the questionnaire two times and 12 for having less than 24 months of postoperative time. In total, 217 patients with at least 2 years of surgery were evaluated.

WR was calculated using both the nadirs postsurgical weight considering a cutoff point of 15% and the percentage of maximum weight loss considering a cutoff point of 20%^4,5^.

## RESULTS

We evaluated 163 women (75.1%) and 54 men (24.9%); 128 (71.9%) of them underwent RYGB and 50 (28.1%) SG. Patients had an average age of 42.2±10.0 years and a postoperative time of 5.2±2.6 years. Weight and BMI before the procedure were 116.4±17.7 kg and 40.0±4.2, respectively. The mean lowest weight achieved was 70.7±14.9 kg after 13.8±7.6 months after the procedure.

The current weight, at the time that the research was performed, was 80.4±18.2 kg, which was 14.0±11.7% greater than the nadir weight. With a cutoff point of 15% of nadir weight, 35% of patients (n=76) regained weight. Values ≥ 20% were used for the cutoff point of WR when the percentage of maximum weight loss was used. Our sample averaged 21.9±17.6% of maximum weight loss, and 46.5% of patients (n=101) obtained values ≥20% (Table 1).

**Table 1:** Analysis of the factors related to weight regain.

	Weight Regain*		p-value
	Yes n=76 (%)	No n=141 (%)	
Preoperative weight in the patient chart (n=180)	109.3 (100.0–126.0)	105.6 (98.0–116.5)	0.045^M^
Preoperative weight referred by the patient	115.0 (107.0–132.8)	112.0 (102.0–122.0)	0.019^M^
BMI before surgery (n=178)	40.1 (39.0–43.0)	39.8 (37.0–41.0)	0.026^M^
Time since surgery (years)	7.0±2.4	4.9±2.4	<0.001^M^
Nadir weight	69.0 (58.0–80.0)	68.0 (60.0–78.0)	0.884^M^
Time after the procedure to nadir (n=213)	12.9±5.7	14.3±8.3	0.455^M^
Current weight	86.5 (77.2–98.2)	72.0 (64.0–84.0)	<0.001^M^
Percentage increase in weight in relation to the lowest weight achieved	22.4 (17.5–31.1)	7.0 (3.8–10.4)	<0.001^M^
Minimum–maximum	15.1–63.5	0.0–14.7	–

M Mann-Whitney U test

* Weight regains considering >15% of nadir weight;

BMI: body mass index.

The preoperative data concerning psychological aspects that were found in the medical charts were as follows: 10 patients used psychiatric drugs, 6.9% had depression, 8.8% binge eating, and 2.7% anxiety.

Regarding the postoperative psychological assessment through the questionnaires BEDS and HADS, 34 patients screened positively for moderate BED and 20 for severe BED, both of which accounted for 24.9% of patients. In total, 26 were screened as light depression, 18 moderate, and 8 severe, which accounts for 24% of the patients. In contrast, when asked about current comorbidities, depression was referred by 43 (19.8%) patients, anxiety by 108 (49.8%), and none referred BED as a current disorder.

WR was significantly associated with greater presurgical weight and BMI, greater postoperative time, and a greater percentage increase in minimum weight (0.045, 0.026, <0.001, and <0.001, respectively) (Table 1). Neither gender, age, surgical technique nor nadir weight was related to a greater WR (p=0.23; 95%CI 0.10–0.13, and 0.88, respectively).

Patients who underwent SG were younger (38.9±10.8 vs. 43.2±9.5; p=0.013) and had a lower BMI before surgery than RYGB [37.0 (34.0–40.5) vs. 40.0 (38.8–42.2); p<0.001]. Although a higher BMI was associated with WR, BMI itself was not associated with either gender or surgical technique. However, men reached the lowest weight faster than women (10.8±5.2 vs. 14.8±8.0; p<0.001). Most of the patients that regained weight were submitted to RYGB, but this was not statistically significant (p=0.13).

No psychiatric diagnosis at preoperative time had a positive association with postoperative WR in either formula (Table 2). Postoperative BED positivity for 24.9% of patients was significantly associated with WR at both thresholds (p=0.004 and p<0.001). Depression screened at the HADS-S questionnaire did not have a positive correlation with WR considering nadir weight (0.521), but current depression and anxiety reported by the patient did show a positive association (p=0.008 and p=0.001). Anxiety reported by the patient impacted WR when the percentage of maximum weight loss was used (p=0.04). Depression almost had a significant relationship (p=0.06) with the same formula. Patients who reported current anxiety and depression are younger (40.4±9.2 vs. 44.1±10.5; p=0.009, p=0.042) and female (p=0.041, p=0.009).

**Table 2:** Psychiatric conditions associated with weight regain

		Weight regain >15% of nadir weight			Weight regain > 20% of percentage maximum weight loss		
		Yes	No	p-value	Yes	No	p-value
Preoperative depression (medical chart)		–	–	**0.63** F	–	–	**>0.99** F
	Yes	4 (26.7)	11 (73.3)	–	7 (6.9)	8 (6.9)	–
	No	72 (35.6)	130 (64.4)	–	94 (93.1)	108 (93.1)	–
Preoperative BED		–	–	**0.67** Q	–	–	**0.425** Q
	Yes	8 (42.1)	11 (57.9)	–	11 (10.9)	8 (6.9)	–
	No	68 (34.3)	130 (65.7)	–	90 (89.1)	108 (93.1)	–
BED		–	–	**0.004** Q	–	–	**<0.001** Q
	Without BED	47 (28.8)	116 (71.2)	–	63 (62.4)	100 (86.2)	–
	Moderate BED	18 (52.9)	16 (47.1)	–	26 (25.7)	8 (6.9)	–
	Severe BED	11 (55.0)	9 (45.0)	–	12 (11.9)	8 (6.9)	–
HADS		–	–	**0.521** M	–	–	**0.585** M
	Normal	54 (32.7)	111 (67.3)	–	73 (72.3)	92 (79.3)	–
	Light	10 (38.5)	16 (61.5)	–	13 (12.9)	13 (11.2)	–
	Moderate	8 (44.4)	10 (55.6)	–	10 (9.9)	8 (6.9)	–
	Severe	4 (50.0)	4 (50.0)	–	5 (5.0)	3 (2.6)	–
Current comorbidities referred by the patient		–	–	–	–	–	–
	Depression	23 (53.5)	20 (46.5)	**0.008** Q	26 (25.7)	17 (14.7)	**0.061** Q
	Anxiety	50 (46.3)	58 (53.7)	**<0.001** Q	58 (57.4)	50 (43.1)	**0.049** Q

Q χ² test

M Mann-Whitney U test

F Fisher’s test;

BED: binge-eating disorder; HADS: Hospital Depression Scale.

Bold indicates statistically significant p-values.

## DISCUSSION

This study has yielded valuable data regarding WR after bariatric surgery. It reinforces previous results that indicate the influence of psychopathologies on bariatric procedures’ outcomes^31^. A Brazilian perspective on weight outcomes and its possible correlations have a great impact on scientific literature since Brazil currently holds second place in the worldwide ranking of bariatric procedures^7^.

This study was performed only with private healthcare system patients. Therefore, results may differ from public healthcare system and international centers due to the different pre- and postoperative guidelines adopted. Such influence can be noticed at the 79.1% of patients submitted to RYGB, while in the public system, SG is the most performed technique^24^. Despite this peculiarity, the sample is a consistent portrait of the population that met criteria to be submitted to the procedure: 75% women of 41 years of age (36–48) with mean preoperative BMI of 40 (37–42). Thresholds for WR were chosen according to the results of King (2018), who compared the performance of the WR formulas with mental health decline and satisfaction with surgery. A 15% of nadir weight and 20% of maximum weight loss performed well with these outcomes (p=0.03 and p=0.09; p<0.008 and p<0.001). The results of 35 and 46.5% of patients in the present study for each formula, respectively, were consistent with the variations of literature: 27–50.2%^22,32^. Greater preoperative weight, BMI, and postoperative time 7.4 (5.6–8.9) were the main weight outcomes to influence WR. Most patients started regaining weight after reaching nadir weight (2–4 years) but continued until 10 years postoperatively with a significant variation in the percentage of weight regained between patients (0–63%). Therefore, it is not possible to say how much weight patients will regain, even though the weight predictors described above may designate that a patient requires a closer, stricter, and longer follow-up^9,16,17,24^.

WR was significantly influenced by both mood and eating pathologies, but some controversies must be discussed. Neither depression nor binge eating preoperatively impacted WR, a topic that needs agreement within the literature^2,9,10,21,23^. These data must be seen cautiously since preoperatively, psychiatric comorbidities found in medical charts were available for only 43 out of 217 patients, with 6.9% (n=15) experiencing depression, 8.8% (n=19) binge eating, and 2.7% (n=6) anxiety. Almost 50% of bariatric surgery candidates have a current or past psychiatric disorder. These data question the low prevalence of preoperative psychiatric disorders among our sample^14^. Since this is documented information, it is possible to question a patient’s failure to report mental illness, either due to embarrassment or due to fear of procedure’s denial. A surgeon’s failure to investigate these comorbidities may also be considered here.

In contrast, postoperative BED was significantly associated with both WR measures (p=0.004 and p<0.001), which confirms data from recent literature^5,10,13,20^. Although some studies have inferred some improvement in BED after the surgery^9,16^, our research found the contradictory results. Since we did not access lifetime eating pathology at baseline, it is not possible to conclude if these 54 patients may have had it before surgery or developed again after the procedure^15,16,29^.

It is important to note that this study only considered the *DSM-V* definition of BED. However, recent studies signal other maladaptive eating behaviors that are generally neither screened nor documented, such as grazing, loss of control eating, picking and nibbling, and night eating^8,17^. Considering these other behaviors, the prevalence of eating disorders could have been much greater than the one found in the present research. These other habits may be equally or more relevant than BED itself postoperatively. As a response to external stressors, eating small portions of high-caloric foods rather than huge amounts still stimulates the brain’s reward system, since these neuropsychiatric pathways are not altered with the surgery^12^. Hanvold et al.^13^ found that smokers had a significantly lower WR, which supports the hypotheses that finding ways to feed neuropsychological pathways is a major cause of WR maintenance^19,30^.

Interestingly, while 24.9% of patients screened positive for binge eating, none of them reported it when asked about current diseases, but instead, 49.8% reported anxiety. A possible hypothesis is that patients may refer to having anxiety rather than BED, because one of the origins of BED comes from anxiety. On the contrary, anxiety itself may be another mood disorder that should be screened and evaluated in future research. As a result, these data bring up the need of making patients aware of the impact of both formal diagnosis and subthreshold maladaptive eating behaviors that can put them at risk for worse outcomes^16^.

As opposed to BED, the understanding of depression’s impact on WR is still controversial^2,16^, despite the evidence that each condition is a risk factor for the future development of the other^6^. Although the results of the HADS-D subscale did not impact WR, possibly due to its screening rather than diagnostic characteristic, the current depression reported by the patient had a significant impact on WR (p=0.008 and p=0.06). Self-reported depression was found mostly in female (p=0.041) and young patients (p=0.042)^6^, supporting the results of King (2019). Depression’s impact on surgical outcomes is not only limited to the statistical results but also it impairs cooperativeness^11^ and impedes motivation, both of which interfere with overall adherence to protocols, diet^4^, physical activity^25^, behavioral change, and long-term follow-up adherence^8^. All of these are considered critical protectors for WR, long-term surgical success, and quality of life^9,31,32^. Therefore, the need for the implementation of a standardized follow-up program, such as the Ontario Bariatric Network, is clear, which includes a strict mental health aid to patients for protocol adherence and behavioral change^1^.

WR often means the return of medical comorbidities that the surgery’s weight loss was able to remit^10,33^, with no significant difference from the preoperative baseline (p=0.67). The confirmation of the rate of WR, its interaction with time, and clinical outcomes shows that it can determine the success of the procedure rather than weight loss alone. Therefore, it should be a major concern for both the surgeon and the patient during follow-ups.

As many patients largely believe that the surgery alone will cure obesity, it is important to raise awareness of patients’ active treatment participation to maintain the weight acquired with surgery, since returning to old habits undermines surgical, anatomical, and hormonal changes. A multidisciplinary team of surgeons, nutritionists, physical educators, psychologists, and psychiatrists specialized in bariatric patients is essential for providing the comprehension of the chronicity of the disease. They are also responsible for supplying patients with the tools necessary to make internal changes to actively maintain their weight loss.

## CONCLUSION

The long-term postbariatric WR data found in the present study highlight the concept that obesity is a chronic and progressive disease that requires specific treatment and constant monitoring. Above all, continuous monitoring should focus on the psychiatric aspects of obesity, and both surgeons and patients should be made aware of the impact of psychopathologies on surgeries’ long-term success.
